# Nutrition for Youth Athletes with ADHD: What We Know and Practical Applications

**DOI:** 10.3390/nu18020282

**Published:** 2026-01-16

**Authors:** Tyler B. Becker, Ronald L. Gibbs

**Affiliations:** 1 Department of Food Science and Human Nutrition, Michigan State University, East Lansing, MI 48824, USA; 2 Michigan State University Extension, Health and Nutrition Institute, Michigan State University, East Lansing, MI 48824, USA

**Keywords:** attention-deficit hyperactivity disorder, sports nutrition, youth athletes

## Abstract

Over 10% of US children and adolescents have attention-deficit hyperactivity disorder (ADHD), with a similar prevalence among youth athletes. While ADHD may confer certain athletic performance advantages such as heightened quickness, decision-making and periods of hyperfocus, it also poses some challenges including reduced concentration, frustration, and possible increased injury risk. Pharmacologic treatments, including stimulant-based medications, can improve attentiveness and athletic performance but could alter nutritional behaviors such as appetite suppression. This paper reviews the current literature on nutritional strategies to provide practical sports nutrition guidelines for children and adolescent athletes with ADHD. Evidence suggests that optimizing energy intake, emphasizing complex carbohydrates, improving fat quality intake, and consuming adequate amounts of micronutrients may support both athletic performance and ADHD symptom management. In contrast, excessive added sugars and saturated fats are associated with poorer outcomes and manifestation of ADHD symptoms. Although no research examining nutritional interventions in youth athletes with ADHD have been performed, applying established sports nutrition principles for youth athletes with ADHD offers a promising approach to enhance performance, reduce injury risk, and support the long-term health of the athlete.

## 1. Introduction

Currently, 10.47% of US children and adolescents have attention-deficit hyperactivity disorder (ADHD) [1]. This is much greater than the global percentage of 7.6% in children and 5.6% in adolescents [2]. The discrepancy in the prevalence of ADHD in the US and other countries could be due to several reasons including differences in the diagnostic criteria used and increased awareness of it [3]. However, the prevalence of ADHD in the population may be greater, as a study examining ADHD symptoms in young adults from the United Arab Emirates reported symptoms suggestive of probable ADHD of 34.7% [4]. It has been estimated that 7–8% of athletes have ADHD [5]. In younger athletes aged 15 to 19 years, this percentage ranges between 4.2% and 8.1% [6]. ADHD is considered a neurodevelopmental disorder where patients display levels of hyperactivity, inattention, and impulsivity [7]. Symptoms of ADHD occur due to imbalances in monoamine neurotransmitters, primarily dopamine, norepinephrine, and serotonin [8]. Among adults with ADHD, the annual total societal excess cost due to the condition is USD 122.8 billion, with excess yearly costs of productivity loss and healthcare services being USD 28.8 billion and USD 14.3 billion, respectively [9]. In children with ADHD, the total yearly societal excess costs associated with the condition are USD 19.4 billion, and USD 13.8 billion among adolescents with ADHD [10]. Provided with these challenges, understanding the effects of the disorder in athletes with ADHD is important.

Having ADHD may provide children and adolescent athletes with some advantages over their counterparts without ADHD [11,12,13]. However, several negative behaviors are associated with this disorder. For example, in a review by White et al. [14], it was identified that athletes with ADHD may manifest the disorder in several ways, including issues with focus and concentration, frustration, an argumentative attitude, and oppositional behavior. Additionally, athletes with ADHD are more likely to become injured compared to their counterparts without ADHD [14]. Furthermore, athletes with ADHD are more likely to have had more concussions and have more severe concussion-related comorbidities compared to their peers without ADHD [6]. This increase in injury risk is hypothesized to be due to symptoms of inattentiveness and impulsiveness [15]. Understanding how nutritional strategies can alleviate ADHD symptoms [16,17] could theoretically reduce injury risk due to these ADHD symptoms.

Currently, 53.6% of children and adolescents, aged 3 to 17 years of age, are taking ADHD medication to treat symptoms of it [18]. Studies examining the effects of ADHD medication (e.g., amphetamines) on performance have been performed for decades [19], with earlier studies indicating improvements in athletic performance [20]. Additionally, a recent systematic review and meta-analysis by Berezanskaya et al. [21] corroborated these findings and found that amphetamines and methylphenidate consistently demonstrated improvements in athletic performance. However, these medications can affect nutritional intake such as decreasing appetite [22], which could lead to negative energy availability and negatively impact athletic performance [23]. Therefore, understanding how these medications can influence athletic performance through interactions with nutritional behaviors is important.

Nutritional strategies have been examined to reduce such symptoms in children and adolescents with ADHD, which could theoretically reduce these symptoms in an athletic setting [16,17]. Additionally, some recommendations have been suggested. In 2022, the American College of Sports Medicine published an article by renowned sports nutrition expert, Nancy Clark, describing nutritional considerations for athletes with ADHD [24]. In this article, Clark highlights some potential differences between athletes with ADHD compared to those without, while also indicating nutritional considerations to have for athletes on ADHD medications. Overall, understanding how nutritional strategies alleviate ADHD symptoms remains understudied regarding sports injury prevention and athletic performance.

Additionally, it is widely known that most children and adolescents do not meet the US Dietary Guidelines for Americans [25]. This is represented by an overconsumption of “unhealthy” foods like sweets and sugary beverages and an underconsumption of more nutrient-dense foods like fruits, vegetables, and whole grains. It has also been shown that children and adolescents with ADHD are more likely to follow this eating pattern compared to non-ADHD youths. For instance, in a case–control study by Salvat et al. [26], it was found that children and adolescents aged 5 to 13 years consumed more simple sugars, consumed less foods rich in key micronutrients, and had overall higher BMI compared to non-ADHD youths. And while efforts to improve diet quality among children and adolescents are aimed to reduce the risk of long-term negative health outcomes, such as increased risk for cardiovascular disease in adulthood, improvements in diet quality may also help improve ADHD symptoms in youths. In a recent review of the literature by Lange et al. [27], the authors examined several cohort studies to determine the adherence of various nutritional strategies and their effects on ADHD symptoms. While there were some promising correlations between adherence to more nutrient-dense dietary patterns (i.e., those high in fruits and vegetables and low in saturated fats and added sugars) and improved ADHD symptoms, there is still much work to be performed in this area.

Overall, there is a need to improve diet quality among children and adolescents. These improvements can help improve long-term health markers, ensure healthy growth and development, and even help reduce the risk of injury among children and adolescent athletes [28]. Further, there is emerging literature to show that these same improvements in diet quality may also improve ADHD symptoms among children and adolescents [16]. Therefore, the purpose of this paper was to provide practical nutritional applications for youth athletes with ADHD after examining the literature.

## 2. Diagnosis of ADHD in Youth

ADHD is diagnosed by examining impairments of two categories: inattention and hyperactivity/impulsivity according to the Diagnostic and Statistical Manual of Mental Disorders, 5th edition (DMS-5) [29]. Six or more symptoms of inattention and/or six or more symptoms of hyperactivity/impulsivity are needed to diagnose children younger than 17 years of age with ADHD [30]. Five or more symptoms of either category are required for diagnosis in adolescents and adults 17 years of age and older. Additionally, these symptoms need to have been present for at least six months and are considered inappropriate for the individual’s developmental level [30]. Further diagnosis requires the conditions to be met in two or more settings, evidence that symptoms negatively affect the individual’s life in some manner, and that the symptoms are not better explained by another mental health disorder. It should be noted that several countries utilize criteria from the International Classification of Diseases, 11th revision (ICD-11) to diagnose ADHD [31]. The ICD-11 uses similar standards for diagnoses with clear differences such as more symptom categories, no clinical threshold for the number of identified symptom categories aside from clinical judgement, and a more flexible age of onset identification.

## 3. Benefits of ADHD for Athletic Performance

It has been hypothesized that children and adolescents with ADHD are more likely to self-select into sports as it provides a positive and reliable environment for managing ADHD symptoms [32]. Indeed, an abstract examining 138 Canadian university students found that there was a higher rate of ADHD in intermediate/high-level athletes than low-level athletes [33]. This is further supported by other evidence illustrating a propensity for individuals with ADHD or symptoms of it gravitating towards sports participation [12,34,35].

This could be due to athletes with ADHD potentially having several athletic benefits over their non-ADHD counterparts [11]. Some athletes with ADHD report impulsivity as a benefit to their sport as it can improve quickness and their ability to be unpredictable to their opponents during a competition [11]. Additionally, this impulsivity could lead to quick decision-making [12]. Athletes with ADHD often report having a “hyperfocus” during their sport, which can block out distractions during competition and practice [13]. This heightened sense of awareness to the skill at hand can thereby improve performance.

## 4. Potential Detriments of ADHD for Athletic Performance

Although having ADHD may offer some benefits during sport, it has been hypothesized that athletes with ADHD are more likely to take risks during practice and competition, thereby increasing their risk of injury [36]. Indeed, research does reveal that adolescents with ADHD are more likely to have had a concussion compared to their peers without ADHD [37]. There are several concussion-related comorbidities in athletes with ADHD including increased concussion risk, lower baseline neurocognitive testing, increased symptomatology, and prolonged recovery following a concussion [6]. However, a recent consortium that examined 1044 concussion cases among collegiate athletes did not notice a significant difference for days until symptom resolutions or days until return to sport between athletes with and without ADHD [38]. Further, a more recent study in adolescents found no differences in total concussion symptoms, symptom severity, and neurocognitive functioning following the concussion or three months post-concussion between those with and without ADHD [39]. It should be noted that athletes with ADHD who are treated with ADHD medications have lower odds of a concussion compared to their non-medicated counterparts [40]. This protective benefit is hypothesized to be due to lower impulsivity and improved executive functioning [30], and could thereby explain the differences in concussion risk [37,39]. Additionally, adolescent athletes with ADHD are more likely to incur a musculoskeletal injury compared to those without ADHD. Among 17,710 baseball players under 25 years of age (average age 17.7 ± 3.93 years), it was found that athletes with ADHD have a greater odds of thorax, abdomen, and pelvic injuries, and ankle sprains compared to players without ADHD [41]. Factors associated with musculoskeletal injuries in children and adolescents with ADHD include comorbid hypertension, substance use disorder, diagnosed thyroid disorder, anxiety, and diabetes status [42]. Therefore, it can be assumed that the condition warrants considerations to reduce injury risk.

A few studies have examined the association of different performance outcomes among adults with diagnosed ADHD [43] or with symptoms of ADHD [44]. Adults with ADHD have a lower estimated VO2max, vertical jump, and a lower number of completed sit-ups for the sit-up test, compared to adults without ADHD [43]. Adults with low grip strength are more likely to have ADHD and have inattention/memory problems than adults with greater grip strength [44]. Additionally, adults with low muscular endurance, tested using push-ups, are more likely to be diagnosed with ADHD, or have inattention/memory problems, and impulsiveness/emotional liability compared to adults with a high muscular endurance. However, these differences could be due to other consequences associated with adults with ADHD including lower educational attainment and lower household income [43]. It is difficult to ascertain if these differences are due to having the ADHD condition or due to the other factors associated with it.

Research examining whether differences in physical performance tests exist between children with ADHD and those without ADHD, having yielded mixed results [45,46]. In a group of 127 children with ADHD and 96 children without ADHD, it was found that children with ADHD performed poorer on the side-to-side jump test than their counterparts without ADHD [45]. In contrast, a study examining 51 elementary-aged students aged 7 to 10 years did not notice a significant difference in push-ups, curl-ups, pull-ups, sit-and-reach test, endurance run, and shuttle run performance tests between students with and without ADHD [46]. This lack of differences in muscular endurance was further corroborated in a study by Kang et al. [47], although they observed that both non-medicated and medicated children with ADHD had lower scores on balance and agility tests compared to children without ADHD. Additionally, a study by Van Riper et al. [48] observed slower response speeds and lower response accuracy in working memory during exercise in adolescents with ADHD. Since the physical performance outcomes tested were different, more research is needed to examine if discrepancies or similarities exist among children and adolescents with ADHD compared to those without.

## 5. Effects of ADHD Medications on Athletic Performance and Considerations

Treatment for ADHD includes medications, behavioral therapy, and physical activity [11]. There are several medication options for ADHD that consist of stimulant medications and non-stimulant medications [5]. Stimulant medications for ADHD include norepinephrine and dopamine reuptake inhibitors (e.g., methylphenidate), selective noradrenaline reuptake inhibitors (e.g., atomoxetine), and alpha2 adrenergic receptor agonists (e.g., guanfacin) [49]. ADHD medications provide several beneficial outcomes for those with ADHD including decreases in mood disorders, substance use disorders, traumatic brain injuries, and other injuries [50]. Additionally, several of these medications have been investigated for their effects on exercise performance, with generally positive results [21,51,52]. However, their use in sport is controversial and raises ethical concerns due to perceived advantages [11]. As a result, medications for ADHD are strictly regulated by sporting bodies including the National Collegiate Athletic Association and the International Olympic Committee [53].

### 5.1. Effects of ADHD Medication on Athletic Performance

Several studies have been conducted examining the impact on adult athletic performance from medication used to treat ADHD going back to the 1950s [20]. Aside from methamphetamine, stimulant-based medications have positive effects on athletic performance such as power output, time to exhaustion, and exercise performance [21]. This is likely due to their ability to increase attentiveness, acting on the central nervous system, and possibly on the peripheral nervous system, thereby providing improvements in performance outcomes. For example, methylphenidate consumption improved time trial time and power output in cyclists performing the trials in a warm condition (30 °C) [54]. This equated to a significant improvement of 16% better times in the time trials. Interestingly, there were no significant differences in trial times nor power output while participants cycled in a lower temperature (18 °C). Additionally, the ratings of perceived exertion were not different between either group during either temperature condition. However, core temperatures did significantly improve in the methylphenidate group while exercising in the warm condition. The researchers speculated that methylphenidate administration inhibited the central nervous system during exercise in warmer conditions, thus reducing fatigue and allowing for a higher power output.

Stimulant-based ADHD medications can also improve strength in adults [51]. Using 15 subjects, King et al. [51] observed that methylphenidate significantly increased mean grip trial force compared to placebo. Additionally, changes were observed in the brain connectivity between the insular cortex and orbital frontal cortex of the brain, suggesting that methylphenidate caused psycho-physiological interactions which occurred simultaneously with increases in grip strength. In addition, some ADHD medications have been hypothesized to reduce fatigue, thereby allowing athletes to perform at a higher level for a given intensity [12]. Thus, it could be suggested that ADHD medications could improve ratings of perceived exertion (RPE). However, a meta-analysis by Berezanskaya et al. [21] did not report a difference in RPE between a placebo and ADHD medication among five studies.

Compared to stimulant-based medication for ADHD, little research has been performed examining the effects of non-stimulant medications for ADHD on exercise performance [55,56]. In an abstract by Fen [55] using 60 adult athletes with ADHD, it was found that groups treated with atomoxetine reported lower ADHD scores after 12 weeks. Additionally, this study also revealed that atomoxetine coupled with featured football teaching also increased exercise performance scores. Cordery et al. [56] found significantly greater improvements in total work performed, in a cycling test among nine women, while taking bupropion compared to placebo. In contrast, another study examining the effects of bupropion on various exercise tests did not observe a change in performance [57]. More research is needed to understand the extent that atomoxetine and other non-stimulant ADHD medications have on performance.

Although most of the research examining ADHD medication benefits in athletics has been examined in adults, some have examined performance effects in children and adolescents [21]. Altszuler et al. [58] observed that methylphenidate significantly improved immediate attention, rule-following, and motor coordination in children aged 5 to 12 years of age. A study among 14 boys (10.9 ± 1.1 years) with ADHD on stimulant medication did not observe a difference during submaximal exercise on VO2, respiratory exchange ratio, and ratings of perceived exertion [59]. However, it was observed that heart rate significantly increased during submaximal exercise while the participants were on medication. It should be noted that the researchers observed physiological changes, but not perceptual changes in the participants at peak exercise, and this was only observed in 6 out of 13 of the participants, suggesting inter-individual differences in responses between participants during exercise.

Inhaled L-methamphetamine in a low- and high-dose condition did not improve miles traveled in a 20 min stationary cycling session in 12 adolescents [52]. In addition, there were no significant measured cardiovascular changes between groups aside from a significant difference in diastolic blood pressure in the low-dose L-methamphetamine condition at 5 min post-ride. This was not observed for the higher dosage compared to placebo at any of the time points post-ride. Thus, the researchers concluded that inhaled L-methamphetamine at low- and high-dosages did not improve cycling performance, and the significant increase in diastolic blood pressure 5 min post-ride in the low-dose condition was too small to indicate biological significance. Similarly, a study examining fitness performance in 70 males aged 7 to 12 years with ADHD did not observe differences in the number of push-ups and sit-ups performed between those with ADHD who take methylphenidate compared to those with ADHD who were non-medicated [60]. In summary, stimulant-based medications for ADHD appear to have more profound effects on athletic performance in adults compared to youths, but more research is needed to confirm these effects.

Additionally, among children and adolescents prescribed ADHD medications, there appears to be a lower risk of unintentional injuries in children and adolescents with ADHD, which may discourage risky behaviors during competition, thereby offering an additional benefit during competition [61]. A large cohort of Danish children found that individuals with ADHD who were treated with ADHD drugs had a reduced risk of injuries by 43% and a reduction in emergency ward visits by 45% compared to their non-medicated peers with ADHD [62].

As previously mentioned, due to the possible performance benefits from these types of medications and potential issues with abuse, the use of ADHD medications in sport is quite regulated [63]. However, it has been argued that banning stimulants in sports for athletes with ADHD does not guarantee fair play [64]. As a result, several sporting organizations have adopted the use of a therapeutic use exemption which offers athletes the option to still compete in their respective sports without penalty resulting from their treatment with ADHD medications [30].

### 5.2. ADHD Medication Considerations

However, emerging research suggests that children with ADHD on ADHD medication may have several sports performance deficits compared to children without ADHD [65]. In a study of 120 children, it was observed that children with ADHD on ADHD medication had lower manual dexterity, ball skills, and balance compared to children without ADHD [65]. Furthermore, medicated children with ADHD reported less participation in individual sport. However, the authors noted that poor aerobic fitness partially explained this lower sports participation in children with ADHD-prescribed ADHD medication.

The use of amphetamine-based ADHD medications is associated with several negative health outcomes including cardiovascular health outcomes [11]. In a systematic review by Nanda et al. [66], the researchers noted a slight increase in BP and heart rate, for those with ADHD taking stimulant medications, but this did not result in increased risk of cardiovascular events, although a longer cumulative duration of ADHD medication is associated with increased hypertension risk and arterial disease [67]. Regardless, it is not recommended that stimulant-based ADHD medications be used in athletes at risk for cardiovascular disease [30]. Common side effects of stimulant-based ADHD medication include a decreased appetite, trouble falling asleep, headaches, and stomachaches [66]. Stimulant medication can also increase the production of stomach acid and thereby increase the risk of gastrointestinal issues [66].

Stimulant-based medications, such as methylphenidate, increase core temperature and heart rate in athletes performing exercise in warm conditions [54], thus increasing their risk for heat-related illnesses. However, a large database study using data from 1,082,112 individuals aged 6–24 with ADHD found that stimulant-based medications actually reduced heat-related illnesses [68]. The authors suggested this result is due to differences in experimental vs. clinical environments. Possible explanations include the possibility that patients with ADHD taking stimulants are better able to control and monitor heat illness symptoms, that stimulant medications increase thirst, thereby reducing dehydration, and that perhaps stimulant use does not negatively influence thermoregulatory mechanisms as once thought.

Muscle pain has been reported with the use of some ADHD medications [69,70]. For example, in a case–control study using 25 adults with ADHD who were responders to methylphenidate, it was found that the adults with ADHD had heightened muscle tone and reported higher pain levels compared to controls [70]. However, more recently it has been suggested that ADHD medications, including stimulants, can be used to alleviate chronic pain by improving cognitive function and addressing central sensitization [71]. Additionally, methylphenidate can raise pain thresholds in children with ADHD to normal levels [72]. At this time, it is prudent to examine muscle pain due to ADHD medication as a case-by-case basis. If a child reports an increased muscle tone and pain levels attributed to the medication, it is suggested to consult with a primary care practitioner to assess the medication type and dosage. Medication type and/or dosage may need to be modified to alleviate symptoms.

## 6. Nutritional Strategies to Alleviate Symptoms in Youth Athletes

To date, no published manuscripts have examined the effects of nutritional interventions, specifically on children and adolescent athletes with ADHD. However, several review articles have been published examining the effects of dietary and nutritional treatments on alleviating ADHD symptoms in children and adolescents [16,17]. Therefore, we will provide practical applications using the existing literature to generate recommendations. These recommendations can be summarized in Table 1.

Medication-naïve children with ADHD have, on average, a resting expenditure that equates to 6.5 kilocalories per kg of fat-free mass higher than children without ADHD [75]. This equates to an extra 325 kcals per day for a 50 kg youth athlete. Interestingly and in contrast, research reveals that children with ADHD who are prescribed stimulant medications have a lower physical activity expenditure, and thus lower energy expenditure (−4% to 8%) during a given day while taking their medication, compared to days when they did not [76]. This is likely attributed to a lower energy expenditure in general, as a result of taking these medications. Therefore, it is suggested that children and adolescent athletes with ADHD and not on ADHD medications should consume slightly more kilocalories than recommended to compensate for this increased energy expenditure [28,77]. This could include slightly more carbohydrates to compensate for the extra energy expenditure resulting from the condition. In contrast, athletes aged 6 to 12 years with ADHD on ADHD medications without reported appetite suppression should consume slightly less kcals (~5%) [76], to offset possible unplanned weight gain. Adolescents with ADHD, regardless if medicated for the condition or not, often have dysregulated appetite hormones including greater levels of fasting insulin and leptin compared to those without ADHD [78]. Leptin acts to suppress hunger and promote satiety [79]. With all of these considerations in mind, it may be prudent to monitor kilocalorie intake and seek consultation with a registered dietitian to make sure the athlete is meeting the kilocaloric needs of their sport.

Children with ADHD often have lower blood glucose levels than those without ADHD [80]; however, some research has revealed a positive correlation with HbA1c levels and having ADHD [81]. Although more research is needed to determine the association with ADHD and blood glucose levels, some research has revealed that a decrease in carbohydrate intake could reduce ADHD symptoms [82,83]. One study with 47 children with ADHD measured a decrease in ADHD symptoms after participants reduced their average daily carbohydrate intake from 206.9 g to 174.6 g per day [82]. It should be noted that there was not a comparison group in that study and that the carbohydrate decrease was attributed to nutritional counseling that instructed children to consume carbohydrates in a range of 45–65% of daily kcals, with daily carbohydrate intake following the intervention being 42.4% of daily kcals. This is only slightly less than the pre-intervention amount of 48% of daily kcals coming from carbohydrates. Since these decreases are not drastic decreases, it is still hypothesized that a more regular consumption of carbohydrates is needed to maintain blood glucose levels [84], and perhaps the type of carbohydrates such as processed sweets are associated with ADHD symptoms instead [85]. Further, it is suggested that a higher consumption of sugar among children with ADHD is a consequence of ADHD and not a determinant [86]. Regardless, recommendations for children and adolescent athletes include adequate carbohydrate consumption, with an emphasis on complex carbohydrates, which are needed to improve performance and reduce fatigue [77]. Carbohydrate recommendations for athletes range from 3–5 g per kg of bodyweight per day on low-intensity days, up to 8–12 g per kg of bodyweight per day on days where moderate–high intensity activity is performed for over a long duration [73].

Reviews that have examined the literature about the relationship of ADHD with nutrition have not provided any protein considerations nor recommendations that would suggest a different need in those with ADHD [17,27]. Therefore, protein recommendations for children and adolescent athletes with ADHD are suggested to follow recommendations for athletes of 1.2–2.0 g per kg of bodyweight per day [73], with 1.5 g per kg of bodyweight per day being considered a good target for youth athletes [74]. In contrast, there may be considerations with fat intake and likelihood of having ADHD and symptoms of ADHD [87,88]. For example, increased intakes of saturated fats are associated with an increased likelihood of being diagnosed with ADHD [87] and children with ADHD have lower levels of *n*-3 polyunsaturated fatty acids (PUFAs), including docosahexaenoic acid (DHA) and eicosapentaenoic acid (EPA) [89]. Several mechanisms have been identified linking a high saturated fatty acid intake with ADHD, including impairment in dopamine function, increased levels of pro-inflammatory cytokines, decreased brain-derived neurotrophic factor levels, and gut dysbiosis [87]. In contrast, *n*-3 PUFAs mechanisms associated with a reduction in ADHD symptoms include improved inflammatory responses, improved hypothalamus–pituitary–adrenal axis stress responses, and a more favorable gut microbiome [89]. As a result, several studies have examined the effects of *n*-3 PUFAs in children and adolescents with ADHD [90,91,92]. A meta-analysis of seven studies by Chang et al. [90] discovered that *n*-3 supplementation improved ADHD clinical symptom scores. However, a recent Cochrane meta-analysis revealed that there was little evidence of *n*-3 PUFA supplementation for reducing ADHD symptoms [93]. Regardless of these mixed results from pooled studies, regular *n*-3 PUFA intake, particularly DHA and EPA, supports neuroprotection by reducing neuroinflammation and reducing cognitive decline with aging [94], rationalizing the regular consumption of them. Furthermore, high *n*-3 PUFA consumption during childhood and adolescence is associated with several positive cardiometabolic outcomes during adulthood [95], suggesting an additional benefit for their consumption. Although the aforementioned experimental studies used supplementation as a treatment, issues with dietary supplement regulation [96] and possible contamination [97] make an emphasis on food sources of these nutrients as a more ethical option in this age demographic. Good food sources of *n*-3 PUFAs to be emphasized in the diet of a child and adolescent athlete with ADHD include walnuts, chia seeds, flaxseed, and salmon, with the latter being high in DHA and EPA [98]. Additionally, other species of fish provide high levels of DHA and EPA including rainbow smelt, lake herring, and whitefish [99]. Since dietary fat recommendations for those with ADHD do not exist, aside from types to prioritize (e.g., PUFAs), the recommendation is to consume 20–35% of one’s daily total energy coming from fat, with no more than 10% of one’s daily total energy coming from saturated fat [74].

Several associations between micronutrient intakes and ADHD diagnosis have been observed with some mechanisms postulated [100,101]. Although it is unclear as to which zinc mechanisms are implicated with symptoms of ADHD, it is involved in several bodily processes including neurotransmitter metabolism and melatonin production that theoretically could increase ADHD symptoms due to inadequate intake [102]. An adequate intake of iron may be needed for reducing ADHD symptoms due to iron’s role in neurotransmitter metabolism along with oxygen transport and deoxyribonucleic acid synthesis [102]. Adequate levels of vitamin D may also be implicated with symptoms of ADHD as it is involved in neurotransmitter regulation [103]. A study examining micronutrient intake and ADHD association used Mendelian randomization with data from major European genome-wide associations and micronutrient concentrations observed a significant association with ADHD and low copper levels, but not magnesium, iron, zinc, selenium, folate, vitamin A, vitamin B12, nor Vitamin D levels [101]. In a case–control study examining nutrient intakes of 146 Egyptian children with ADHD, compared to 146 community controls without ADHD, it was observed that lower intakes of zinc, copper, iron, magnesium, and potassium were associated with having ADHD [100]. In contrast, higher intakes of sodium were associated with having ADHD. In a similar study using 68 children with ADHD and 68 controls without ADHD, it was found that serum levels of copper, magnesium, and zinc were 21%, 4%, and 7% lower in the children with ADHD compared those without [104]. As a result, several studies have examined the effectiveness of micronutrient supplementation on reducing ADHD symptoms. In a sample of unmedicated children aged 6–12 years with ADHD, Johnstone et al. [105] observed that supplementation with a micronutrients supplement led to improvements in ADHD symptoms following 8 weeks of supplementation. When examining single micronutrient supplementation on ADHD symptoms in children and adolescents with ADHD, research has primarily been performed examining the effects of zinc, iron, magnesium, and vitamin D [17,27]. A systematic review of zinc and iron supplementation and ADHD by Granero et al. [102] examined five studies that supplemented with zinc, two studies that supplemented with iron, and two additional studies that supplemented with both. The researchers at baseline noted that low zinc and/or iron levels were associated with higher ADHD symptoms, and that supplementation with either appeared to improve ADHD severity, although the effect sizes were quite low. In a study of 66 Iranian children with ADHD, supplementation of magnesium (6 mg/kg of bodyweight per day) and vitamin D (50,000 IU/week) led to improvements in conduct, social problems, and anxiety/shy scores [106].

Similarly to suggestions for *n*-3 PUFA intake, the recommendation is to emphasize food sources for these micronutrients of interest. Good food sources of zinc include oysters, crabmeat, and ground beef, while good food sources of iron include dark meat poultry, organ meats such as liver, spinach, and cashews [107]. Foods high in magnesium include cod, lentils, and almonds. Aside from sun exposure, good food sources of vitamin D include fatty fish and fortified foods such as milk [107].

## 7. Other Nutritional Considerations for Youth Athletes Prescribed ADHD Medication

It is important to note that ADHD medications, particularly stimulant medications, can reduce appetite [22], and thus children and adolescent athletes on these medications may have difficulty meeting nutrition recommendations for sport [77,108]. Clark suggests consuming a large breakfast prior to taking the medication and to swap lower kilocalorie drinks with higher kilocalorie versions in an effort to increase kilocalorie intake [24].

The relationship of stimulant-based ADHD medications increasing risk of heat-related illnesses is mixed [54,68]. To err on the side of caution, general recommendations for fluid requirements are advised for youth athletes [73], with coaches and athletes alike monitoring their fluid intake and loss through monitoring body mass loss [109]. Before exercise, athletes should consume fluid volumes equivalent to 5–10 mL per kg of bodyweight two to four hours prior to [73]. During exercise, the recommendation is to consume approximately 0.4 to 0.8 L of fluid per hour of exercise, which is dependent on several factors including environmental conditions and intensity and duration of the exercise [73]. During exercise, athletes may lose body water via 0.3 to 2.4 L of sweat, which also includes salts, potassium, calcium, and magnesium [110]. As a result, after exercise, athletes should consume approximately 125–150% of fluid compared to the amount of bodyweight lost [73].

## 8. Conclusions

Youth athletes with ADHD represent a distinct subset of athletes in which neurodevelopmental differences overlap with the demands of sport and nutrition. While ADHD can provide certain competitive advantages, it also carries some risks and could increase injury risk. Medications to reduce symptoms of ADHD may improve performance and reduce injury risk, but they also introduce nutritional challenges such as appetite suppression, altered energy expenditure, and potential gastrointestinal or cardiovascular concerns. Although no studies have directly examined nutritional interventions in children and adolescent athletes with ADHD, the existing literature on nutritional associations and ADHD and nutritional interventions in individuals with ADHD provide valuable insights. Evidence suggests that optimizing energy intake, emphasizing complex carbohydrates, ensuring adequate protein, emphasizing *n*-3 PUFAs, and consuming adequate amounts of some micronutrients (e.g., zinc, iron, magnesium, vitamin D) may support both athletic performance and symptom management.

These conclusions emphasize the significance of individualized nutritional strategies to meet the demands of sport and ADHD management. Coaches, parents, and other practitioners should recognize that nutrition is not only a tool for enhancing performance and recovery but also to alleviate ADHD-related symptoms. There is some emerging research in this area. For example, Sogard and Mickelborough [111] have suggested the use of caffeine to help alleviate symptoms of ADHD due to its ability to increase alertness and concentration. Supplementation of the gut microbiome with specific bacteria strains is also an emerging area of interest as research has revealed differences in the composition of the microbiota in those with ADHD compared to those without [112]. In addition to dietary supplementation to alleviate ADHD symptoms, some research has suggested modification of the entire diet to improve symptoms. One 12-week trial of children with ADHD observed a significant decrease in ADHD symptoms following the Dietary Approaches to Stopping Hypertension diet [113]. Future research should directly investigate the impact of targeted nutritional interventions in children and adolescent athletes with ADHD, bridging the gap between clinical insights and practical sports nutrition applications.

## Figures and Tables

**Table 1:** Nutritional Recommendations for Children and Adolescent Athletes with ADHD.

Dietary Variable	Recommendation	Notes
Daily Kilocalorie Intake	Amount needed to meet the demands of activity while supporting growth and development [28]	Consider impact of stimulant medication on energy needs
Carbohydrate	3–5 g per kg of BW; up to 8–12 g per kg of BW [73]	Daily amounts are determined by type of activity, intensity, and duration
Protein	1.5 g per kg of BW [74]	Emphasize lean sources
Fat	20–35% of daily kilocalories <10% from saturated fat [74]	Emphasis on good sources of PUFAs, specifically EPA and DHA

Abbreviations: ADHD, attention-deficit hyperactivity disorder; BW, bodyweight; DHA, docosahexaenoic acid; EPA, eicosapentaenoic acid; PUFA, polyunsaturated fatty acid.

## Data Availability

No new data were created or analyzed in this study; data sharing is not applicable to this article.

## Abbreviations

The following abbreviations are used in this manuscript:

ADHD	Attention-deficit hyperactivity disorder
BMI	Body mass index
DHA	Docosahexaenoic acid
DSM-5	Diagnostic and Statistical Manual of Mental Disorders, 5th edition
EPA	Eicosapentaenoic acid
ICD-11	International Classification of Diseases, 11th revision
PUFA	Polyunsaturated fatty acid
RPE	Rating of perceived exertion
US	United States
VO2max	Maximal volume of oxygen consumption
